# Long-term effects of biochar application on the growth and physiological characteristics of maize

**DOI:** 10.3389/fpls.2023.1172425

**Published:** 2023-06-14

**Authors:** Mengfei Cong, Yang Hu, Xia Sun, Han Yan, Guangling Yu, Guangmu Tang, Shuhuang Chen, Wanli Xu, Hongtao Jia

**Affiliations:** ^1^ College of Resources and Environment, Xinjiang Agricultural University, Urumqi, China; ^2^ Xinjiang Key Laboratory of Soil and Plant Ecological Processes, Xinjiang Agricultural University, Urumqi, China; ^3^ Institute of Soil and Fertilizer and Agricultural Sparing Water, Xinjiang Academy of Agricultural Science, Urumqi, China; ^4^ Key Laboratory of Saline-alkali Soil Improvement and Utilization (Saline-alkali Land in Arid and Semi-Arid Regions), Ministry of Agriculture and Rural Affairs, Urumqi, China

**Keywords:** biochar, aeolian sandy soil, antioxidant enzymes, maize yield, long-term effect

## Abstract

Biochar, as a soil conditioner, has been widely used to promote the growth of maize, but most of the current research is short-term experiments, which limits the research on the long-term effects of biochar, especially the physiological mechanism of biochar on maize growth in aeolian sandy soil is still unclear. Here, we set up two groups of pot experiments, respectively after the new biochar application and one-time biochar application seven years ago (CK: 0 t ha^-1^, C1: 15.75 t ha^-1^, C2: 31.50 t ha^-1^, C3: 63.00 t ha^-1^, C4: 126.00 t ha^-1^), and planted with maize. Subsequently, samples were collected at different periods to explore the effect of biochar on maize growth physiology and its after-effect. Results showed that the plant height, biomass, and yield of maize showed the highest rates of increase at the application rate of 31.50 t ha^-1^ biochar, with 22.22% increase in biomass and 8.46% increase in yield compared with control under the new application treatment. Meanwhile, the plant height and biomass of maize increased gradually with the increase of biochar application under the one-time biochar application seven years ago treatment (increased by 4.13%-14.91% and 13.83%-58.39% compared with control). Interestingly, the changes in SPAD value (leaf greenness), soluble sugar and soluble protein contents in maize leaves corresponded with the trend of maize growth. Conversely, the changes of malondialdehyde (MDA), proline (PRO), catalase (CAT), peroxidase (POD) and superoxide dismutase (SOD) manifested an opposite trend to the growth of maize. In conclusion, 31.50 t ha^-1^ biochar application can promote the growth of maize by inducing changes in its physiological and biochemical characteristics, but excessive biochar application rates ranging from 63.00-126.00 t ha^-1^ inhibited the growth of maize. After seven years of field aging, the inhibitory effect of 63.00-126.00 t ha^-1^ biochar amount on maize growth disappeared and changed to promoting effect.

## 1 Introduction

Aeolian sandy soil is one of the most important reserved, cultivated land resources in arid and semi-arid areas. But due to its disadvantages of having low nutrient content and poor water retention, crops planted in it face adverse environmental effects (Baiamonte et al., 2020; Jahromi et al., 2020; Ibrahim et al., 2021), which leads to poor crop growth and low yield. Maize (*Zea mays* L.), one of the world’s most important food crops, is mainly planted in semi-arid areas (Ul-Allah et al., 2015). But the Aeolian sandy soil limits the growth of maize (Yan et al., 2022). Alternatively, biochar as soil conditioner produced from organic waste under pyrolysis process, improve aeolian sandy soil by increasing its nutrient content and water-holding capacity (Kammann et al., 2015; Hussain et al., 2017). Biochar has been shown to improve soil quality by altering soil structure, increasing water-holding capacity, etc. (Han et al., 2020; Hossain et al., 2020). Additionally, biochar can enhance soil fertility by maintaining nutrients and stimulating microbial activities (Minhas et al., 2020). Therefore, biochar application in aeolian sandy soil may help solve the underutilization of aeolian sandy soil.

At present, numerous studies reported the biochar application in soil. For example, the prominent improvement in plant roots traits, leaf area, plant growth, morphological and yield-related parameters were observed with addition of biochar at 2 and 4 t ha^-1^, while, plant height, number of grains per cob, grains and biological yield decreased with biochar addition 6 t ha^-1^ (Minhas et al., 2020). Some studies have also found that biochar can significantly increase the chlorophyll content of maize, while was adverse effects when the application rate exceeded 12 t ha^-1^ (Khan et al., 2022). However, there is still a lack of research on the effects of biochar on plant physiological and biochemical characteristics. Environmental stress can lead to high accumulation of hydrogen peroxide and superoxide (reactive oxygen species, ROS) in crops, which can be harmful to important biomolecules (such as lipids, proteins, pigments and nucleic acids), ultimately inhibiting crop growth and yield (Ashraf, 2009; Golldack et al., 2014; Noctor et al., 2014; Vwioko et al., 2019). However, plants can upregulate their antioxidative defense mechanism by stimulating the activities of key antioxidative enzymes, including superoxide SOD, CAT, and POD, to counteract ROS (Ashraf, 2009). SOD converts superoxide ions into hydrogen peroxide (H_2_O_2_) and oxygen (O_2_) (Zhang et al., 2004; Wang et al., 2018). Subsequently, CAT and POD break down H_2_O_2_ into H_2_O and O_2_ (Foyer and Halliwell, 1976; Wang et al., 2018). Previous studies have found that biochar can scavenge reactive oxygen species by activating the production of antioxidant enzymes (Haider et al., 2022; Mahmoud et al., 2022). For instance, plants treated with 0.75% biochar experienced less oxidative stress due to stimulated activity of the antioxidant defense systems (Abideen et al., 2020). Besides the scavenging effect of ROS, osmotic adjustment (such as proline, soluble sugar, and soluble protein) is broadly recognized as providing high-energy reactions to maintain cell turgor, which is necessary for crop growth (Hare et al., 1998). The application of biochar has been shown to improve the synthesis of stressed proteins and proline in plants, thereby maintaining the osmotic protectant and osmotic potential of plants under environmental stress (Haider et al., 2022; Mahmoud et al., 2022). While it is clear that antioxidant enzymes and osmoregulators play an important role in crop growth, there is still a lack of research on them, which limits the in-depth study of the physiological and biochemical characteristics of maize under biochar application. A meta-analysis of worldwide research to evaluate the impact of biochar found that the biochar application results vary with raw materials used, pyrolysis temperature, soil properties, and climate (Jeffery et al., 2016). Therefore, the impact of biochar on plant growth is still questionable.

It is worth noting that most of the current studies on the effects of biochar on maize growth are conducted in short-term experiments (within 1 year) (Yeboah et al., 2016; Minhas et al., 2020). Only a few studies reported the effects of biochar on crops after the addition of biochar for several years or more. For instance, adding 31.5-47.25 t ha^-1^ biochar for five years promoted the uptake of soil phosphorus by maize straw (38.6-71.3%) and grain (20.9-25.5%) (Cao et al., 2020). Moreover, one-time application of biochar can enhance the growth and yield of wheat and maize for four years (Hu et al., 2021). However, several studies have demonstrated that crop growth and nutrient uptake were only promoted after many years of biochar application. For example, when 8 or 20 t ha^-1^ of biochar was applied, the maize yield did not change in the first year post-application, but increased in the next three years (Major et al., 2010). Therefore, there is a need to investigate the long-term effects of biochar on crops, particularly in poor farmland soils such as aeolian sand soil, where the benefits of biochar may be more significant. However, the effects of long-term application of biochar on maize growth, physiological and biochemical characteristics in poor farmland soil, especially in aeolian sand soil, have not been adequately studied.

In conclusion, although some long-term experimental results have reported the long-term effects of biochar, there is still a lack of enough long-term experiments worldwide, especially considering the wide diversity of both biochar and soil, and the results varying with different regions and different crops. Moreover, the physiological mechanism of maize growth under the condition of biochar application is still unclear. Therefore, we used two groups of pot experiments, one for the new biochar application, the other for the one-time biochar application seven years ago, to study the effect of biochar application on maize growth and its after-effect, which could fill the gaps and provide data support for the biochar application in aeolian sandy soil in arid areas of Northwest China. Considering that aeolian sand soil is known to have poor nutrient content (Baiamonte et al., 2020), while biochar is rich in carbon sources and nutrients (Kammann et al., 2015; Hussain et al., 2017) and can remain in soil for hundreds of years (Kuzyakov et al., 2009). We hypothesized that: (1) The growth of maize will still be promoted seven years after the application of biochar. (2) Biochar application can improve the growing environment of maize and reduce the activity of antioxidant enzymes. Therefore, our aims are (a) to analyze and compare the short- and long-term effects of biochar on maize growth and yield in aeolian sandy soil; (b) to identification direct or indirect soil and plant physiological characteristics affecting maize yield; and (c) to reveal the optimal application rate of biochar in aeolian sandy soil.

## 2 Materials and methods

### 2.1 Experimental design

**Figure 1:**
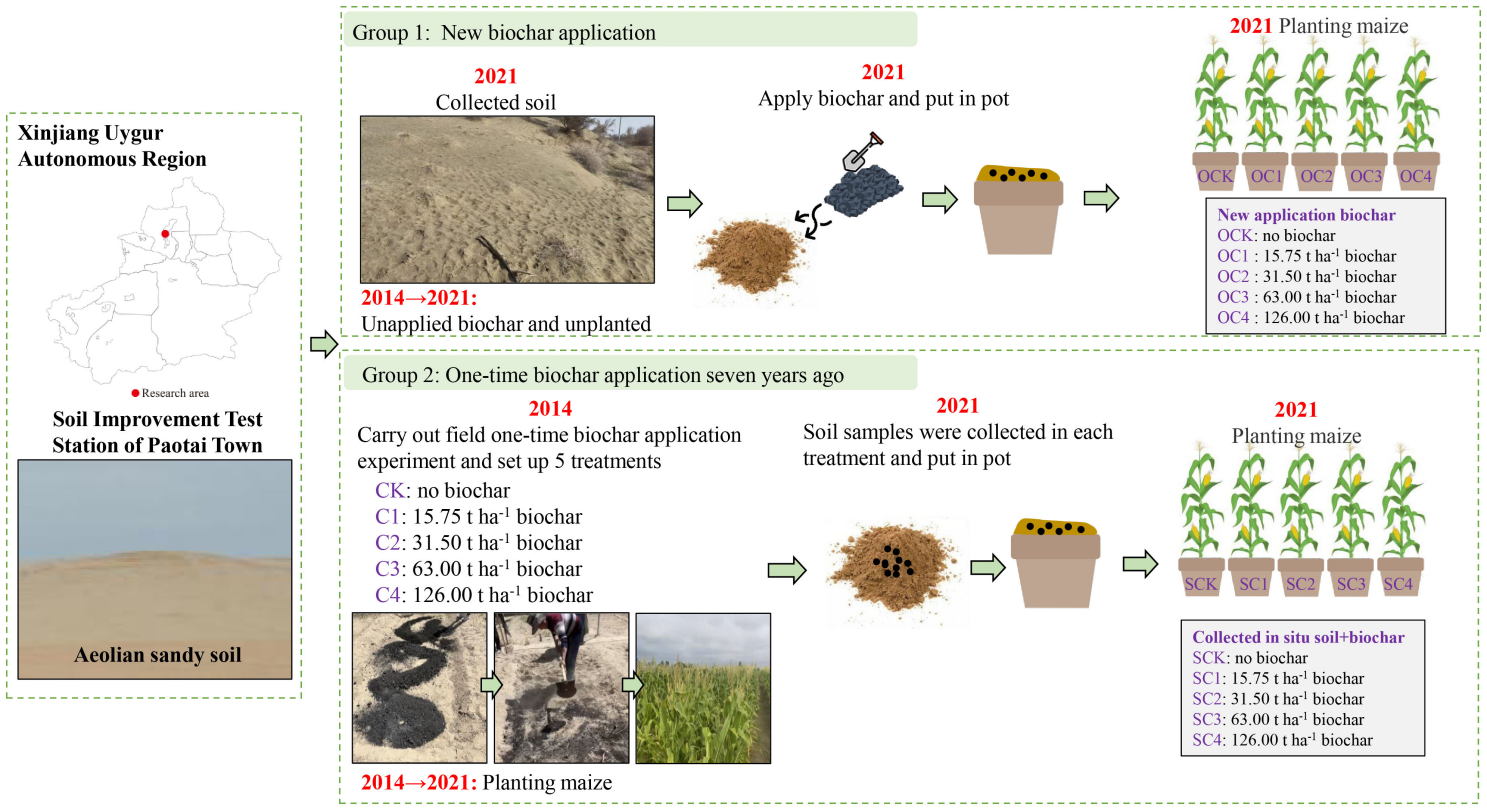
Schematic diagram of test design.

In 2021, two groups of pot experiments were set up using the pot design method in Xinjiang Agricultural University, respectively denoted as Group 1 and Group 2. One group involved new biochar application treatment (Group 1). In 2021, we collected the aeolian sandy soil without biochar application and planting crops, and brought it back to the experiment site for new application of biochar and planting maize (*Zea mays* L.) (**Figure 1**). The second group involved one-time biochar application seven years ago treatment (Group 2). In 2014, we carried out a field application of biochar. In 2021 (seven years later), we collected the *in-situ* aeolian sandy soil from each treatment in the field experiment, and brought it back to the experiment site to be directly potted and planted with maize (*Zea mays* L.) (**Figure 1**).

#### 2.1.1 One-time biochar application seven years ago (Group 2)

Firstly, a field *in-situ* experiment of biochar application was carried out in the Soil Improvement Test Station of Paotai Town, Shihezi City, Xinjiang Uygur Autonomous Region, China. The experiment began in 2014 using a randomized block design with five treatments based on the amount of straw harvested in one year after carbonization (2.625 t ha^-1^): no biochar treatment (CK), one-time application of 15.75 t ha^-1^ (C1), 31.50 t ha^-1^ (C2), 63.00 t ha^-1^ (C3), and 126.00 t ha^-1^ (C4) biochar treatment. Three replicates were set for each treatment, resulting in a total of 15 plots with an area of 4.6 m×7.0 m. The biochar was obtained from wheat straw carbon, carbonized at 450°C for 5 h, crushed, and screened using the 2 mm size. In 2014, before sowing, the biochar was applied in a one-off manner, and mixed evenly with 0~20 cm soil. The basic chemical properties of the aeolian sandy soil and biochar were shown in **Table 1**. Seed sowing was scheduled in May and harvest was arranged in every September. The planted maize variety was “Xinyu No.53”, the irrigated via under-membrane drip irrigation, with is one crop per year. Prior to sampling (2021), field management practices, fertilization and irrigation remained consistent across all treatments for seven years, as indicated in **Table S1**.

**Table 1:** Chemical properties of soil and biochar in the two experiments before sowing.

Treatment	pH	Electrical conductivity (mS cm^-1^)	Organic Carbon (g kg^-1^)	Available nitrogen (mg kg^-1^)	Available phosphorus (mg kg^-1^)	Available potassium (mg kg^-1^)
Soil						
Field experiment in 2014	8.21	—	1.38	7.40	4.60	97.00
Field experiment in 2021	8.19	0.11	6.70	8.21	15.41	92.92
Pot experiment in 2021	8.22	0.12	1.28	7.63	4.96	96.31
Biochar						
Field experiment in 2014	9.7	7.21	660	74.27	80.13	1157
Pot experiment in 2021	9.9	7.22	670	70.44	82.2	1590

Secondly, in 2021 (seven years later), before sowing, the five soil subsamples at 0-20 cm were collected from each plot using a shovel according to the zigzag pattern. And the 15 subsamples from each treatment were mixed as composite samples to produce five combination samples. Subsequently, the soil samples of five treatments were evenly packed into pots (25 cm in diameter and 25 cm in height, without reapplication of biochar), with 15 pots for each treatment (five stages × three replications), resulting in total of 75 pots. The five treatments were denoted as SCK, SC1, SC2, SC3 and SC4, as listed in **Table 2**. The basic chemical properties of the aeolian sandy soil under SCK were as follows in **Table 1**. During sowing, five seeds were planted in each pot, with the extra seedlings removed after the emergence of the seedlings, leaving only one plant in each pot. The planted maize variety was “Xinyu No.53”.

**Table 2:** Application amount of biochar.

Experiment	Treatment	Application amount (t ha^-1^)
New biochar application treatment	OCK	0
	OC1	15.75
	OC2	31.50
	OC3	63.00
	OC4	126.00
One-time biochar application seven years ago treatment	SCK	0
	SC1	15.75
	SC2	31.50
	SC3	63.00
	SC4	126.00

#### 2.1.2 New biochar application (Group 1)

In 2021, aeolian sandy soil without biochar application and without crops was collected in the Soil Improvement Test Station of Paotai Town, Shihezi City, Xinjiang Uygur Autonomous Region, China. The basic chemical properties of the aeolian sandy soil were as follows in **Table 1**. The texture and chemical properties of the sandy soil were basically consistent with those of the 2014 field experiment.

The amount of biochar used in the pots was calculated based on the amount applied in the *in situ* field experiment (Group 2). Firstly, the amount of biochar calculated for each treatment was evenly mixed separately with aeolian sandy soil. Secondly, the mixed samples were put into the corresponding pots (25 cm in diameter and 25 cm in height) according to five treatments. The 15 pots were set up for each treatment (five stages × three replications), resulting in total of 75 pots. The five treatments were denoted as OCK, OC1, OC2, OC3 and OC4, as listed in **Table 2**. The biochar was obtained from wheat straw carbon, carbonized at 450°C for 5 h, crushed, and screened using the 2 mm size. The basic chemical properties of the biochar were as follows in **Table 1**, and the basic chemical properties of soil after new mixture of biochar were shown in **Table S2**. During sowing, five seeds were planted in each pot, with the extra seedlings removed after the emergence of the seedlings, leaving only one plant in each pot. The planted maize variety was “Xinyu No.53”.

All local field management practices were followed consistently across all treatments, including the periods and amounts of fertilizer and irrigation. Detailed information is provided in **Table S1**.

### 2.2 Sampling and measurements

#### 2.2.1 Plant height

At the second leaf (V2) stage (19 days after sowing), sixth leaf (V6) stage (50 days after sowing), tassle (VT) stage (89 days after sowing), blister (R2) stage (108 days after sowing) and black layer (R6) stage (137 days after sowing), three maize plants were selected for each treatment, and the plant height (cm) was measured with steel tape. The height from the base of the maize stem to the highest point of the plant is defined as plant height.

#### 2.2.2 Biomass, nutrient uptake and yield

At the V2, V6, VT, R2, R6 stage, the roots, leaves and stems of three maize were harvested, and their dry weight (DW) was recorded after drying at 75°C until reaching constant weight, which was biomass (g plant^-1^ DW). After that, the roots, stems and leaves were thoroughly ground for nutrient content determination. The roots, stems and leaves were digested with H_2_SO_4_-H_2_O_2_, and the nitrogen content (mg kg^-1^) was determined by Nessler’s reagent (K_2_HgI_4_) colorimetric method, the phosphorus content (mg kg^-1^) was determined by vanadium-molybdenum-yellow colorimetry, and then determined by spectrophotometry (Shimadzu UV-1780, Japan). The potassium content (mg kg^-1^) by flame photometer (Shanghaiyuefeng FP6400, China) (Bao, 2000). Nutrient uptake amount (mg plant^-1^ DW) is the product of nutrient content and biomass. At the R6 stage, three maize seeds in each treatment were collected, dried at 75°C to constant weight, and the weight was recorded. Hundred-kernel weight, spike length (cm), the number of rows per ear and number of grains or kernels per row were also determined.

#### 2.2.3 Leaf greenness, physiological parameters and biochemical parameters

At the V2, V6, VT, R2 stage, the leaf greenness (SPAD readings) of each maize plant leaf (the first leaf in the uppermost part was fully expanded in V2 and V6 stage, and ear-leaf were selected in VT and R2 stage) was measured using the SPAD502 Chlorophyll meter (TOP Cloud- Agri SpAD-502, China).

At the same time, each maize plant leaf (the first leaf in the uppermost part was fully expanded in V2 and V6 stage, and ear-leaf were selected in VT and R2 stage) were destructively collected. After that, it was wrapped in tin foil and placed in a liquid nitrogen tank at -80°C for measuring biochemical parameters.

The content of fresh leaves (FW) soluble protein (μg g^-1^ FW) was estimated following Spector (1978) using Coomassie bright blue G-250 reagent, and the absorbance was recorded at 595 nm using bovine serum albumin as standard. Meanwhile, the anthrone reagent was used to determine the soluble sugar (%) of fresh leaves, and absorbance was measured at 625 nm as described by the method of Irigoyen et al. (1992).

The content of fresh leaves proline (PRO, μg g^-1^ FW) was determined based on the method by Bates et al. (1973). In brief, 0.5 g of the fresh leaf sample was mixed with 3% sulfosalicylic acid. Then, the 2 mL mixture was reacted with glacial acetic acid (2 mL) and acid ninhydrin (2 mL) in a test tube, and the mixture was incubated for 30 min at 100°C in a water bath. After incubation, 5 mL of toluene was added to the reaction combination, and maintained in the dark at room temperature for 20 min to permit separation of the toluene layer from the aqueous solution. The absorbance of toluene later was measured at 520 nm using a spectrophotometry (Shimadzu UV-1780, Japan).

The content of fresh leaves malondialdehyde (MDA, μg g^-1^ FW) in terms of thiobarbituric acid reactive substances (TBARS) was assessed based on the method illustrated by Du and Bramlage (1992). In brief, the MDA was extracted from 0.5 g of the fresh leaf sample with 0.1% trichloroacetic acid (TCA), and the homogenate was centrifuged for 15 min. Subsequently, the resulting supernatant was combined with 20% TCA (4 mL) containing 0.5% TBA, and the mixture was incubated at 95°C for 30 min. After centrifugation for 15 min, the absorbance of mixture was measured at 532 and 600 nm using a spectrophotometry (Shimadzu UV-1780, Japan).

The fresh leaves were first crushed and homogenized in phosphate buffer (5 mL, 50 mmol L-1 phosphate buffer, containing 1 mmol L^-1^ EDTA, 1 mmol L^-1^ phenylmethylsulfonyl fluoride, and 1% polyvinylpolypirrolidone). The resulting homogenized sample was then centrifuged for 30 min to obtain an enzyme extract. Subsequently, the activity of superoxide dismutase (SOD, Unit mg^-1^ protein) was determined based on the method by Beauchamp and Fridovich (1971). For this, the enzyme extract was mixed with a reaction mixture (containing 150 mmol L^-1^ K-phosphate, 13 mmol L^-1^ methionine, 75 µmol L^-1^ p-nitrobluetetrazolium chloride, 2 µmol L^-1^ riboflavin, and 0.1 mmol L^-1^ EDTA), and the SOD was measured at 560 nm using a spectrophotometry (Shimadzu UV-1780, Japan).

The activity of Catalase (CAT, Unit mg^-1^ protein) was anticipated by assessing the prime speed of H_2_O_2_ defeat. Briefly, 100 µL of enzyme extract was added to K phosphate buffer (1.5 ml, 50 mmol L^-1^) and H_2_O_2_ (1.5 ml, 10 mmol L^-1^), and the enzyme activity was measured at 240 nm for 2 min using the method of Yang et al. (2011).

The activity of Peroxidase (POD, Unit mg^-1^ protein) was determined using o-phenylenediamine as a chromogenic indicator in the presence of H_2_O_2_ and enzyme extract, and the absorbance was measured at 417 nm using the method of Vetter et al. (1958).

#### 2.2.4 Soil parameters

Soil pH was measured at a water/soil ratio of 2.5:1 using a pH meter (Mettler Toledo FE28-Standard, Switzerland). Soil electric conductivity (mS cm^-1^) measured by electric conductivity meter (Keruiyongxing DDS-11A, China). Soil organic carbon content (g kg^-1^) was determined by the H_2_SO_4_–K_2_Cr_2_O_7_ external heating method (Bao, 2000). Available N (mg kg^-1^) was determined by the alkali diffusion method. Soil available phosphorus (mg kg^-1^) was extracted by NaHCO_3_ and then determined by spectrophotometry (Shimadzu UV-1780, Japan) (Bao, 2000). Available potassium (mg kg^-1^) was extracted by CH_3_COONH_4_ and then determined by flame photometer (Shanghaiyuefeng FP6400, China) (Bao, 2000).

### 2.3 Statistical analysis

The data was collected in Excel 2018. One-way ANOVA was performed using SPSS 22.0. LSD was used to test the significance (*P*<0.05). Structural equation model (SEM) was used to revealed the direct and indirect effects of biochar on maize yield. In brief, a Z-score transformation was used to standardize all data (Maestre et al., 2012), and take the average of these transformed values as the variables in SEM, where osmoregulators contained soluble sugars and soluble proteins; the soil properties contained pH, organic carbon, available nitrogen, available phosphorus and available potassium; and the ROS contain SOD, POD, and CAT. The structural equation modeling (SEM) analysis was performed using SPSS Amos 24 (IBM, United States). Origin 2018 was used to draw point-and-line figures and bar figures, and R software was used to draw correlation matrix figures.

## 3 Results

### 3.1 Effects of biochar application on plant height, biomass and nutrient uptake of maize

**Figure 2:**
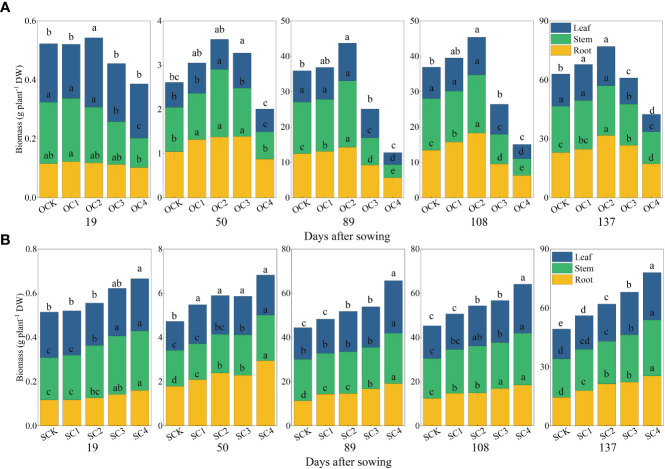
Effect of biochar application on maize plant height. **(A)** New biochar application treatment. **(B)** One-time biochar application seven years ago of treatment. The bar graph in the line chart shows the average plant height of five stages. Same small letter indicates no significance within same experiment at *P*=0.05.

**Figure 3:**
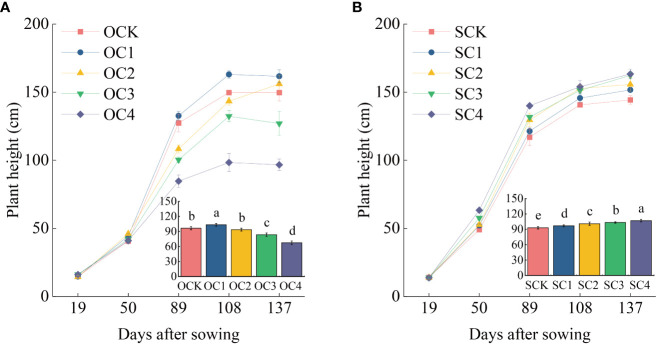
Effect of biochar application on the biomass of roots, stems and leaves. **(A)** New biochar application treatment. **(B)** One-time biochar application seven years ago of treatment. Same small letter indicates no significance within same experiment at *P*=0.05.

In the new biochar application treatment, the OC1 treatment significantly increased plant height of maize by 7.09% compared with OCK, while the OC3 and OC4 treatments significantly decreased by 13.31% and 30.14% (**Figure 2A**). The OC1 and OC2 treatments significantly increased biomass of roots, stems and leaves compared with OCK, while the OC3 and OC4 treatments significantly decreased (**Figure 3A**). In the one-time biochar application seven years ago treatment, plant height of maize was significantly increased by 4.13~14.91% in all treatments with biochar application (SC1, SC2, SC3, and SC4) compared with SCK (**Figure 2B**). Moreover, the biomass of roots, stems and leaves showed a significantly increase with the increasing biochar application (**Figure 3B**).

In the new biochar application treatment, the OC2 treatment significantly increased uptake amounts of nitrogen and phosphorus (20.81% and 36.34%, respectively) compared with OCK. However, the uptake amounts of nitrogen and phosphorus in OC4 treatment was significantly decreased by 19.99% and 19.64% compared with OCK. Meanwhile, the uptake amounts of potassium in OC1, OC2 and OC3 treatments was increased by 16.14~52.42% compared with OCK (**Table 3**). In the one-time biochar application seven years ago treatment, the SC1, SC2, SC3 and SC4 significantly increased uptake amounts of nitrogen, phosphorus and potassium (nitrogen increased by 14.08~59.23%, phosphorus increased by 59.06~117.81%, potassium increased by 30.30~90.93%) compared with SCK (**Table 3**).

**Table 3:** Effects of biochar application on maize nutrient uptake amounts.

Experiment	Treatment	Nitrogen uptake amounts (mg plant^-1^ DW)	Phosphorus uptake amounts (mg plant^-1^ DW)	Potassium uptake amounts (mg plant^-1^ DW)
New biochar application treatment	OCK	1081.96 ± 56.01b	287.12 ± 24.15b	1506.43 ± 101.21c
	OC1	1127.75 ± 121.00ab	330.53 ± 21.66b	1749.63 ± 126.32b
	OC2	1307.12 ± 91.25a	391.45 ± 18.15a	2296.17 ± 335.80a
	OC3	1188.11 ± 35.6ab	325.92 ± 23.74b	2016.03 ± 305.14ab
	OC4	865.68 ± 26.15c	230.73 ± 4.44c	1573.17 ± 73.83c
One-time biochar application seven years ago treatment	SCK	902.33 ± 51.75d	194.64 ± 60.13d	1080.64 ± 47.51d
	SC1	1029.37 ± 41.77c	258.58 ± 27.19cd	1408.03 ± 70.00c
	SC2	1104.54 ± 55.02bc	309.59 ± 25.27c	1521.82 ± 116.65bc
	SC3	1228.87 ± 62.84b	363.60 ± 20.51b	1717.24 ± 98.25b
	SC4	1436.75 ± 39.83a	423.94 ± 24.85a	2063.27 ± 67.07a

Same small letter indicates no significance within same experiment at P=0.05.

### 3.2 Effects of biochar application on physiological characteristics of maize

In the new biochar application treatment, the OC4 treatment significantly decreased SPAD readings of maize leaves at the 50, 89 and 108 days after sowing (decreased by 11.08~30.58%) compared with OCK (**Figure S1A**). Conversely, in the one-time biochar application seven years ago treatment, the SC4 treatment significantly increased SPAD readings of maize leaves at the 89 and 108 days after sowing (increased by 6.78~12.25%) compared with SCK (**Figure S1B**).

**Figure 4:**
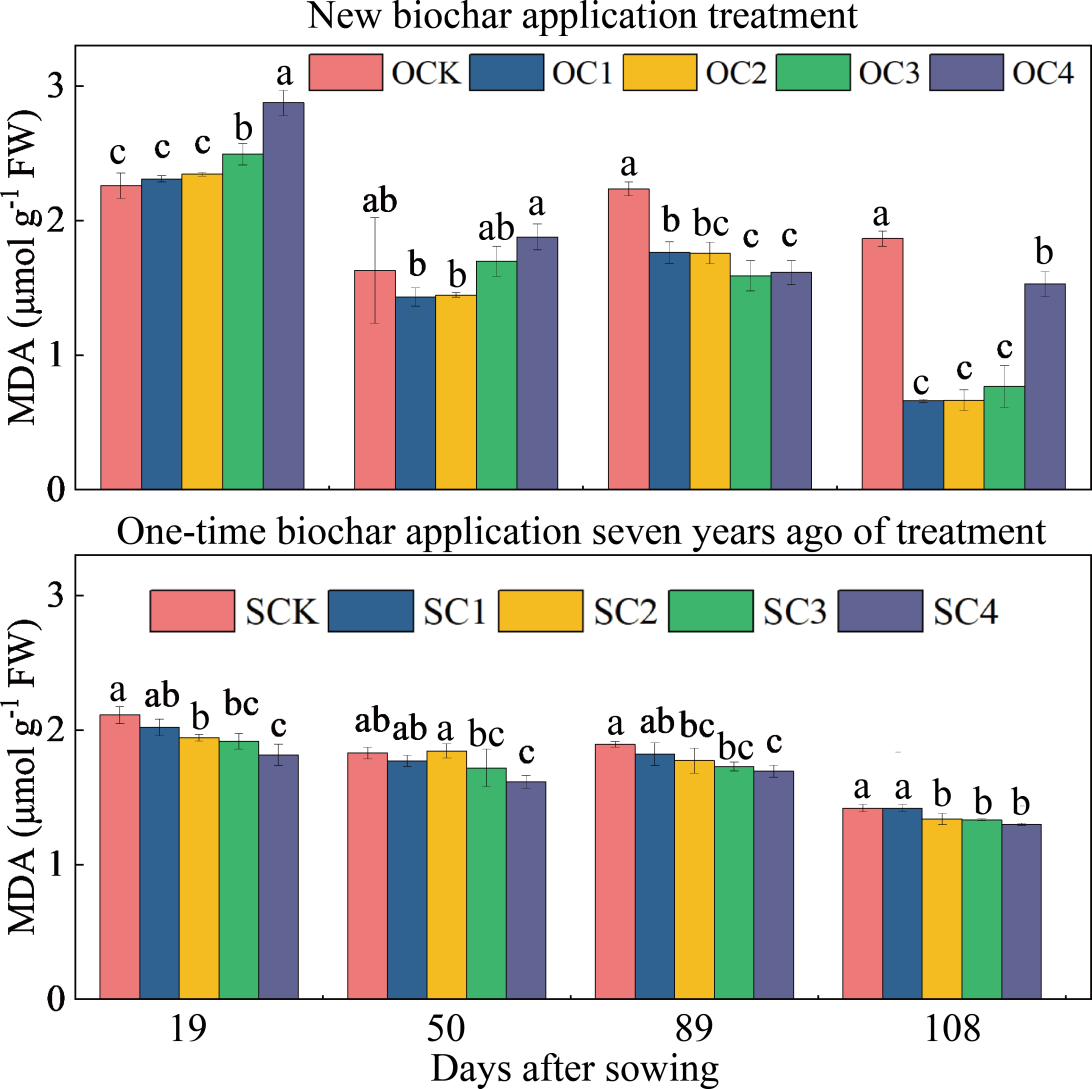
Effects of biochar application on malondialdehyde (MDA) contents in maize leaves. Same small letter indicates no significance within same experiment at *P*=0.05.

In the new biochar application treatment, the OC3 and OC4 treatments significantly increased MDA content at the 19 days after sowing (increased by 10.42~27.33%) compared with OCK, and the OC1, OC2, OC3, and OC4 treatments significantly decreased at the 89 and 108 days after sowing (decreased by 18.11~64.52%) (**Figure 4A**). In the one-time biochar application seven years ago treatment, the SC2, SC3, and SC4 treatments significantly decreased MDA content at the 19, 50, 89 and 108 days after sowing (decreased by 5.67~14.04%) compared with SCK (**Figure 4B**).

**Figure 5:**
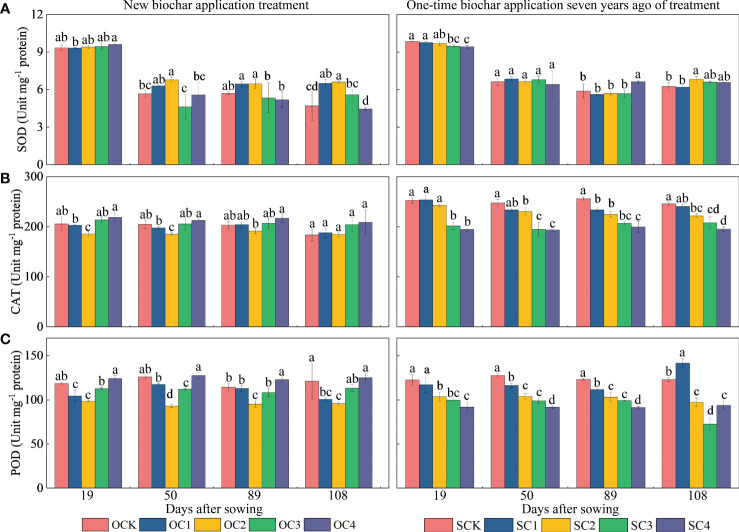
Effect of biochar application on **(A)** superoxide dismutase (SOD), **(B)** catalase (CAT) and **(C)** peroxidase (POD) activities in maize. Same small letter indicates no significance within same experiment at *P*=0.05.

For ROS activity (**Figure 5**), in the new biochar application treatment, the OC2 treatment increased SOD activity at the 50 and 108 days after sowing (13.05~38.44%) compared with OCK (**Figure 5A**). However, the same treatment significantly decreased CAT and POD activity at the 19 and 50 days after sowing (9.60~26.22%), and also significantly decreased POD activity at the 89 and 108 days (16.58%~21.10%) compared with OCK (**Figures 5B, C**). In the one-time biochar application seven years ago treatment, the SC3 and SC4 decreased SOD activity at the 19 days after sowing (3.69~4.33%) compared with SCK. However, the SC4 treatment significantly increased SOD activity at the 89 days after sowing (12.73%) compared with SCK (**Figure 5A**). Furthermore, the SC2, SC3, and SC4 treatments significantly decreased CAT and POD activities at the 19, 50, 89, and 108 days after sowing (15.50~40.97%) compared with SCK (**Figures 5B, C**).

**Figure 6:**
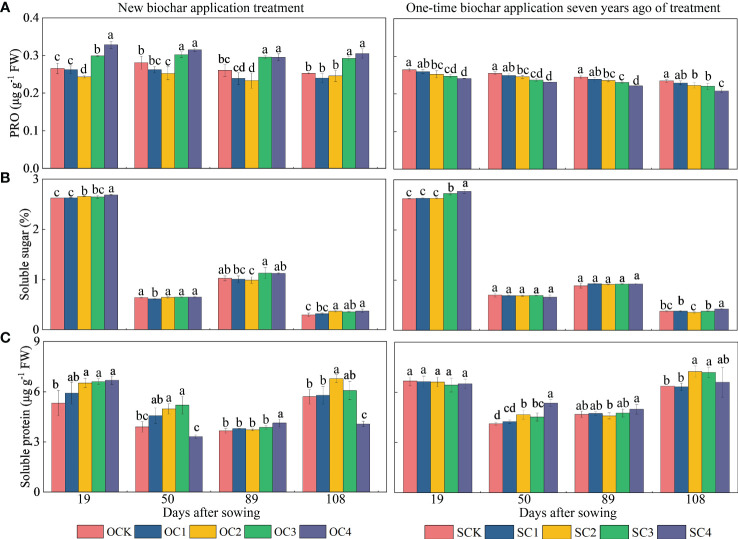
Effects of biochar application on **(A)** proline, **(B)** soluble sugar and **(C)** soluble protein contents in maize leaves. Same small letter indicates no significance within same experiment at *P*=0.05.

For osmoregulators, in the new biochar application treatment, the OC2 treatment significantly decreased PRO content (2.73~10.60%) compared with OCK, while OC3 and OC4 treatments increased at the 19, 50, 89 and 108 days after sowing (7.66~23.75%) (**Figure 6A**). Furthermore, the OC2, OC3 and OC4 treatments significantly increased soluble sugar content at the 19 and 108 days after sowing (0.84~26.03%) compared with OCK (**Figure 6B**). Additionally, the OC3 and OC4 treatment significantly increased soluble protein content at the 19 and 50 days after sowing (24.17~33.40%) compared with OCK (**Figure 6C**). Regarding the one-time biochar application seven years ago treatment, the SC2, SC3 and SC4 treatment significantly decreased PRO content at the 19, 50, 89 and 108 days after sowing (3.48~11.41%) compared with SCK (**Figure 6A**). Moreover, the SC4 treatment increased soluble sugar content at the 19 and 108 days after sowing (5.56~12.50%) (**Figure 6B**), and SC2 and SC3 treatment increased soluble protein content at the 60 and 108 days after sowing (9.79~13.74%) compared with SCK (**Figure 6C**).

### 3.3 Effects of biochar application on maize yield and its components

In the new biochar application treatment, the OC2 treatment significantly increased maize seed yield and spike length (increased by 8.46% and 9.52%, respectively) compared with OCK, while the OC4 treatment significantly decreased (decreased by 31.29% and 10.58%, respectively) (**Table 4**). Both the OC1 and OC2 treatments significantly increased the hundred-kernel weight (increased by 30.28~38.56%) compared with OCK, while the OC4 treatment decreased (decreased by 13.78%) (**Table 4**). In the one-time biochar application seven years ago treatment, all of the SC1, SC2, SC3, and SC4 treatments significantly increased the seed yield of maize (increased by 33.25~62.94%) compared with SCK (**Table 4**). The SC1, SC3, and SC4 treatments significantly increased hundred-kernel weight (increased by 7.83~16.05%) compared with SCK, while the SC1, SC2, and SC3 significantly increased the number of grains or kernels per row (increased by 11.09~15.86%). The SC4 treatment significantly increased the spike length (increased by 21.03%) compared with SCK (**Table 4**).

**Table 4:** Effects of biochar application on maize yield and its components.

Experiment	Treatment	Seed yield (g plant^-1^)	Hundred-kernel weight (g)	The number of rows per ear	The number of grains or kernels per row	Spike length (cm)
New biochar application treatment	OCK	45.41±1.25b	15.82±1.70c	13.67±0.58a	21.33±0.58a	16.07±0.06b
	OC1	45.96±4.46ab	20.61±0.79ab	12.67±0.58ab	22.67±0.58a	18.57±1.37a
	OC2	49.25±0.65a	21.92±0.12a	12.00±1.00b	22.67±2.89a	17.60±0.36a
	OC3	46.89±0.61ab	19.60±1.11bc	12.33±0.58ab	22.67±1.15a	15.63±0.55bc
	OC4	31.20±0.72c	13.64±0.71d	12.00±1.00b	23.00±1.00a	14.37±0.78c
One-time biochar application seven years ago treatment	SCK	39.15±3.48d	20.94±1.44c	12.67±0.58ab	21.00±1.00c	16.17±1.26b
	SC1	52.17±1.60c	23.42±1.06ab	11.33±0.58b	24.00±1.00ab	17.67±0.58b
	SC2	54.96±0.28bc	21.68±0.74bc	13.33±1.15a	23.33±1.15ab	17.20±1.11b
	SC3	57.08±0.16b	24.30±1.03a	12.67±0.58ab	24.33±2.08a	17.10±0.90b
	SC4	63.79±1.89a	22.58±0.64ab	14.00±2.00a	22.00±1.00bc	19.57±0.74a

Same small letter indicates no significance within same experiment at P=0.05.

### 3.4 Relationship between physiological indexes and the growth and yield of maize

**Figure 7:**
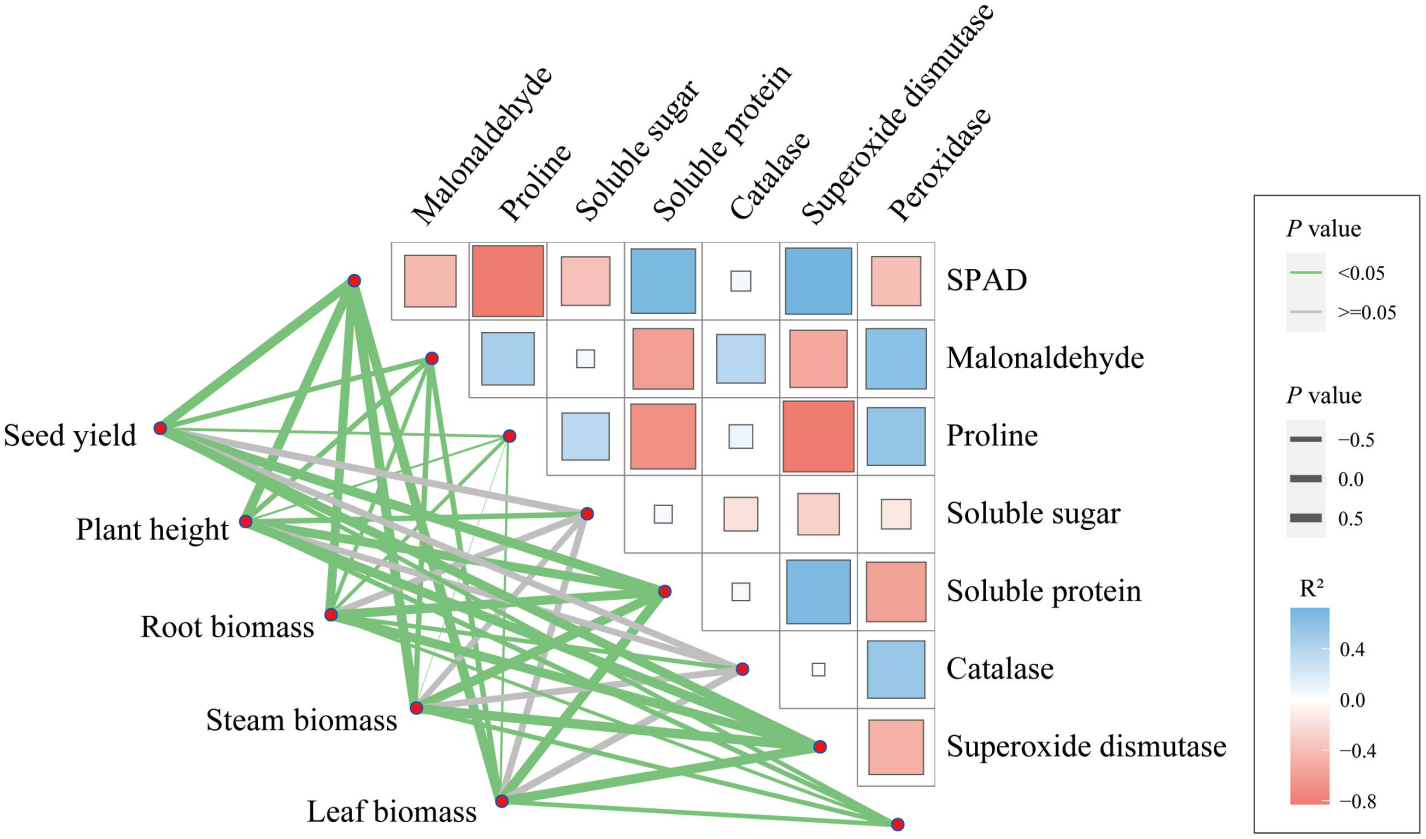
The relationship among yield, growth, and physiological characteristics of maize.

The maize seed yield is mainly significantly related to hundred-kernel weight, ear rows number and spike length (**Figure S2**). Among the constituent factors, the hundred-kernel weight was significantly correlated with spike length, and the number of ear rows was significantly correlated with the number of grains or kernels per row. Furthermore, there is a significant relationship between maize seed yield, growth index, and leaf physiological characteristics (**Figure 7**). Among them, the maize seed yield was significantly correlated with the contents of SPAD readings, MDA, PRO, soluble protein, and activities of CAT and SOD in leaves. Additionally, there is also a certain correlation between the physiological indexes of maize (**Figure 7**).

**Figure 8:**
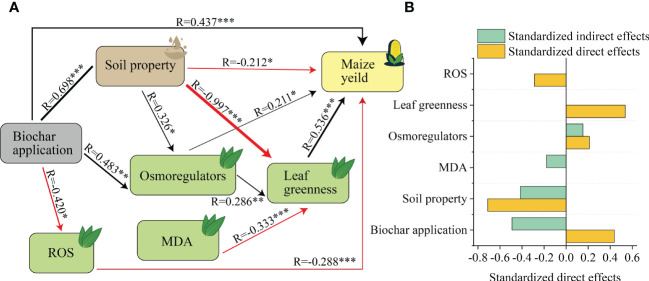
The direct and indirect effects of biochar on maize yield. **(A)** Structural equation model (SEM) revealed the direct and indirect effects of biochar on maize yield. χ^2 = ^10.81, *DF*=9, *P*=0.289. Among them, width of the arrow head indicates the strength of the relationship, Black arrows indicate a significant positive relationship, and red arrows indicate a significant negative relationship (*P* < 0.05), Non-significant paths have been deleted, * indicates *P <*0.05, ** indicates *P* < 0.01. *** indicates *P* < 0.001. **(B)** Standardized effects of biochar addition, physiological characteristics and soil property on maize yield. Osmoregulators contain soluble sugars and soluble proteins, Soil properties contain pH, organic carbon, available nitrogen, available phosphorus and available potassium. ROS contain SOD, POD, and CAT.

SEM revealed direct and indirect effects of biochar on maize yield, showing that application of biochar had a significant positive effect on maize yield directly, and also indirectly through soil properties, osmoregulators and ROS (**Figure 8A**). The highest standardized effect of biochar application and soil properties on yield was observed (**Figure 8B**).

## 4 Discussion

Biochar is mainly composed of carbon and ash, with carbon accounting for the highest proportion (70~80%), and the ash comprises some mineral components like K, Ca, Na, Mg, etc. (Lehmann and Joseph, 2009). By returning biochar to the field, we can return most of the nutrients like Ca, Mg, K, and P to the soil, thereby making it consistent with the sustainable utilization of crop waste (Liang et al., 2006; Cheng et al., 2008). A meta-analysis found that the combined utilization of low-carbon biochar and sandy or acidic soil showed greater potential to improve plant productivity (Dai et al., 2020). This is expected to solve the problem of aeolian sandy soil application. In this study, we found that the biochar application of 31.50 t ha^-1^ significantly promoted the growth of maize (**Figures 2A**, **3A**), making it consistent with numerous reports. E.g., The biochar application significantly promoted the maize seed yield, with the application rate of 5 t ha^-1^ being better than that of 2.5 t ha^-1^ (Yeboah et al., 2016). This is due to: (i) While biochar may reduce the soil bulk density and increase the soil’s total porosity due to its porous structure and large specific surface area, it also improved the root system development, enhanced the plant nutrient uptake ability, and promoted the growth and yield of maize (Oguntunde et al., 2008; Ibrahim et al., 2021). The application of biochar in soil can also improve soil structure, promote the agglomeration of soil mineral particles, and enhance the stability of aggregates (Liu et al., 2014; Dong et al., 2016). (ii) Most biochar is made from crop stalks, so it contains a lot of nutrients. A large number of studies have confirmed that the application of biochar can significantly increase the content of nutrients in the soil, thus affecting the growth of crops (Liang et al., 2006; Cheng et al., 2008; Lehmann and Joseph, 2009; Rogovska et al., 2016; Amin, 2018). Our results also confirmed that the application of biochar significantly increased soil nutrient content (**Tables S2**, **S3**). At the same time, biochar can (a) reduce the leaching of soil nitrate-nitrogen, (b) improve the soil’s nitrate carrying capacity, and (c) reduce the soil nitrate reductase activity, soil denitrification intensity, and soil nitrogen oxide flux, so as to slow down the loss of soil nitrate-nitrogen, thereby maintaining the nitrogen use efficiency of crops, and promoting the increase of plant biomass (Steiner et al., 2008; Zhou et al., 2011; Cao et al., 2019; Liu et al., 2021). (iii) Biochar can also affect microbes, on the one hand, biochar can be a habitat for microorganisms due to its porous structure (Quilliam et al., 2013). On the other hand, biochar application can also affect soil microbial activity and reshape microbial community structure (Ahmad et al., 2016). Thirdly, biochar application also significantly affected the relationship between microorganisms, enhanced the connection between bacteria-fungal communities (Ma et al., 2019). However, microbial changes can affect soil characteristics, such as decomposition of organic matter and regulation of soil carbon dynamics and nutrient cycling, which further affect plant growth (Zheng et al., 2019). These results contribute to our understanding of the ecological effects of biochar application in soil, and further demonstrate that biochar can change the physical, chemical and biological properties of soil and affect crop growth. Our study confirms that biochar significantly affects maize yield by affecting soil chemical properties (**Figure 8**). However, the current researches on the effects of biochar on maize growth and physiology are still limited. The change of crop physiological characteristics is very important in the process of crop growth (Gupta et al., 2020). Therefore, it is necessary to further study the physiological characteristics of maize by biochar, especially in the widely distributed aeolian sand soil.

Aeolian sandy soil forms an unfavorable environment for plant growth due to its low nutrient content and poor water retention ability. Analyzing the plant responses post biochar improvement of soil is necessary for understanding the physiological basis for improving yield and physiological stability (Afshar et al., 2016). Osmoregulation is an important plant physiological mechanism for resisting stress. Organisms can regulate their cellular osmotic balance via accumulation and synthesis of osmoregulators (soluble sugar, proline, and soluble protein) during their normal metabolic process, so as to alleviate the stress-induced damage in plants (Hare et al., 1998). In this study, the contents of soluble sugar and soluble protein in maize leaves increased significantly post biochar application, but the contents of proline decreased significantly (**Figure 6**). Therefore, these results indicated that biochar application changed the osmoregulators in maize leaves to adapt to the soil environmental changes. This is consistent with the results of previous studies. For example, Yildirim et al. (2021) found that the proline content of plants increased significantly under drought stress, but decreased significantly after the application of biochar. Moreover, our previous study at the same sites showed that the application of biochar to aeolian sandy soil significantly increased the soil moisture content, field capacity, and saturated water content due to the unique structure of biochar (Yan et al., 2022), which is supported by a large number of other studies (Busscher et al., 2010; Laird et al., 2010; Ajayi and Horn, 2016; Ajayi et al., 2016). Proline accumulation is considered to be one of the responses of plants to reduce damage under water shortage conditions (Anjum et al., 2011). This suggests that the application of biochar may decreases the proline content of maize leaves by improving soil moisture content and helping plants adapt to environmental changes. Previous studies found that biochar application decreased proline content and increased the relative water content of plants (Nehela et al., 2021). Thus, the positive effect of biochar on plants may be attributed to maintaining water uptake (Naeem et al., 2017) and significantly increasing the relative water content of leaves (Tanure et al., 2019; Ran et al., 2020; Nehela et al., 2021). We believe that the application of biochar in this study affects the water absorption in maize by changing the osmoregulators in maize leaves, indirectly increasing the yield of maize, and also indirectly affecting osmoregulators through changes in soil properties, ultimately impacting maize yield (**Figure 8**).

When plants are under stress, the amount of internal ROS increases sharply. If these ROS cannot be removed in time, they cause serious damage to plants (Shoukat et al., 2019). ROS affects many cellular functions by damaging nucleic acids, oxidizing proteins, and causing lipid peroxidation (Foyer and Noctor, 2005). Plants have a series of coping mechanisms under stress. SOD, POD, and CAT are the main ROS-scavenging enzymes in plants. Their synergistic effect can prevent the ROS-induced damage of plant cell membranes (Foyer et al., 1994; Jithesh et al., 2006). As the final end-product of membrane lipid peroxidation, the MDA content represents the degree of membrane peroxidation and is often used to evaluate the degree of cell membrane damage (Yildirim et al., 2021). In this study, the activities of SOD, POD, and CAT enzymes in maize leaves decreased significantly after application of biochar (**Figure 5**). This is consistent with our second hypothesis. Therefore, this shows that the biochar application alleviates the inhibitory effect of aeolian sandy soil on maize. This is consistent with previous research results. For example, it has been reported that the activities of CAT, POD, and SOD and MDA content of plants in biochar-treated soil have decreased (Farhangi-Abriz and Torabian, 2017). Biochar application can improve the biomass of *Paragonimus carinii* in arid habitats by improving its water status, photosynthesis and antioxidant enzyme activities (Abideen et al., 2020). Under drought conditions, ROS production increases in plants, and increased ROS levels damage plants by causing lipid peroxidation, protein oxidation, enzyme inhibition, chlorophyll degradation, and cell death (Sahin et al., 2018). Biochar application reduces ROS content (**Figure 5**), which has a positive impact on crops. This is consistent with our analysis that the negative effect of biochar application on ROS significantly increased maize yield (**Figure 8**).

In addition, it is important to reveal the long-term effects of biochar. The aging of biochar changes its surface functional groups, physical structure, and even elemental composition, and with the destruction of the microstructure of biochar, the adsorption of trace elements in the soil is also weakened (Naisse et al., 2015). Since the effects of biochar gradually disappear with soil leaching, biochar should be repeatedly applied at certain intervals to maintain its repair effect (Chen et al., 2016). However, the aging of biochar in different soil types needs to be determined. During the initial stage, biochar itself can be used as fertilizer for providing nutrients to plants. However, this effect gradually disappeared over time due to subsequent plant uptake and leaching. Since, biochar can exist in soil for thousands of years (Kuzyakov et al., 2014), a single application of biochar may achieve long-term effects. Additionally, the aging of biochar in soil helps to improve plant nutrient utilization and thereby promote plant growth. While aging in soil, the surface modification of biochar can improve its potential to retain nutrients, making it easily absorbable by plants (Cheng et al., 2006), which can further improve crop yield over time (Crane-Droesch et al., 2013). Our study results also found that biochar still promoted maize growth and yield, even seven years after being applied in the soil (**Figures 2B**, **3B**). This is consistent with our first hypothesis. Some studies have found that the biochar application significantly increased the maize yield (increased by 9.4~35.5%) within eight years (Zhang et al., 2021). This helps us explain the long-term effects of biochar on maize growth and yield.

It is worth noting that in our study, the maize growth was inhibited by a high amount of new biochar application (126.00 t ha^-1^) in aeolian sandy soil. This inhibition could be due to multiple reasons. Firstly, the aeolian sandy soil in this study belongs to the alkaline soil, and the pH and electrical conductivity of biochar is also high. Therefore, the application of a large amount of biochar will significantly increase the pH and electrical conductivity of soil and inhibit the uptake to nutrients and growth of crops (Yuan and Xu, 2011; Jin et al., 2022). This was also confirmed by our results, which showed that high application significantly increased soil pH (**Table S2**). Secondly, since biochar is produced by high-temperature pyrolysis under oxygen limiting conditions, it may contain pollutants that can induce phytotoxicity and cytotoxicity (Hu et al., 2021). And biochar may negatively impact the beneficial soil microbial communities (Mukherjee and Lal, 2014). And, fresh biochar due to its negatively charged surface can adsorb cationic nutrients (Yao et al., 2012), leading to their incomplete utilization by plants, which may negatively impact plant growth (Kammann et al., 2015). Our results also found that the application of biochar indirectly reduced maize yield through a positive effect on soil (**Figure 8**).

But after seven years of biochar application, the same amount promoted the growth and yield of maize, thus showing that the toxic effect of biochar dissipates with increasing time. First, this may be due to the decrease in pH of biochar during the natural aging process, which alleviates the phenomenon of high soil pH caused by large amounts of alkaline biochar application (Wang et al., 2020). Second, it may be that during natural aging, soil minerals accumulate on the surface of biochar, forming an organic-mineral complex, blocking cracks and channels on the surface of biochar, reducing its adsorption capacity and enabling plants to effectively absorb nutrients (Wang et al., 2021). It is also worth noting that the biochar amended soil may have suffered a dilution effect due to tillage and field management practices during the seven years. Consequently, the actual application rates of biochar in the new biochar application treatment may have been higher than that in the one-time biochar application seven years ago treatment, which could have contributed to the differences observed between the short-term and long-term experiments.

Multiple studies conducted in different regions of the world have found that the biochar application may produce different results, which are closely related to raw materials, pyrolysis temperature, soil properties, and climate (Jeffery et al., 2016). Therefore, further research is needed to verify and optimize the technology of biochar application, to achieve its potential benefits on plant growth and yield.

## 5 Conclusion

In summary, the application of biochar was found to have a significant impact on maize yield by altering soil properties and maize physiological characteristics, both directly and indirectly. Specifically, moderate biochar application (15.75~31.50 t ha^-1^) promoted maize yield by increasing SPAD, soluble sugar and protein content and decreasing malondialdehyde, PRO and ROS, while single application of the high amount of biochar (63.00~126.00 t ha^-1^) would have a negative impact on maize growth. Furthermore, the inhibitory effect of high amount of biochar application on maize growth changed to a promotive effect after seven years of aging in the field. However, this is closely related to the raw materials of biochar and the properties of the soil where it is applied. The long-term effects of different biochar in different soil types need to be further studied. Furthermore, comparing field-aged biochar with fresh biochar will help understand the potential mechanism behind its potential benefits.

## Data availability statement

The original contributions presented in the study are included in the article/**Supplementary Material**. Further inquiries can be directed to the corresponding authors.

## Author contributions

MC, HY, and XS conceived and designed the study, and wrote the manuscript. MC, YH, XS, GY, HY, GT, WX, and HJ were responsible for performing the field and laboratory work. MC, YH, and HJ analyzed the data. All authors contributed to the article and approved the submitted version.
