# Supplementary material for: Long-term effects of biochar application on the growth and physiological characteristics of maize
Source: Front Plant Sci. 2023 Jun 14;14:1172425. doi: 10.3389/fpls.2023.1172425 (PMC10319354; doi:10.3389/fpls.2023.1172425)
Supplement: Supplementary file 1 [file DataSheet_1.docx]

**Supplementary Material**

TABLE S1 Irrigation and fertilization

|  | Emergence (VE) | Sixth leaf (V6) | Tenth leaf (V10) | Twelfth leaf (V12) | Tassle (VT) | Silking (R1) | Blister (R2) | Milk  (R3) | Dough (R4) | Total |
| --- | --- | --- | --- | --- | --- | --- | --- | --- | --- | --- |
| Volume of irrigation (m^3^/ha) | 163.6 | 600 | 600 | 600 | 600 | 600 | 600 | 563.6 | 472.8 | 4800 |
| Amount of fertilizer applied | | | | | | | | | | |
| N (kg/ha) | 27 | 78 | - | - | 91 | - | 52 | - | 248 | 258 |
| P_2_O_5_ (kg/ha) | 14 | 38 | - | - | 45 | - | 26 | - | 123 | 123 |
| K_2_O (kg/ha) | 8 | 25 | - | - | 32 | - | 16 | - | 81 | 81 |

TABLE S2 Effect of new biochar application on soil properties

| **Index** | **Treatment** | **days after sowing** | | | | |
| --- | --- | --- | --- | --- | --- | --- |
|  |  | **19** | **50** | **89** | **108** | **137** |
| pH | OCK | 8.22±0.02e | 8.21±0.02d | 8.24±0.03d | 8.23±0.02d | 8.22±0.10c |
|  | OC1 | 8.32±0.01d | 8.32±0.01c | 8.32±0.07cd | 8.31±0.03c | 8.35±0.03b |
|  | OC2 | 8.37±0.02c | 8.37±0.03c | 8.38±0.04bc | 8.34±0.01c | 8.37±0.03b |
|  | OC3 | 8.61±0.03b | 8.56±0.06b | 8.46±0.03b | 8.44±0.02b | 8.43±0.04b |
|  | OC4 | 8.88±0.04a | 8.84±0.06a | 8.83±0.09a | 8.80±0.07a | 8.85±0.02a |
| Organic Carbon (g/kg) | OCK | 9.28±0.07d | 9.35±0.06c | 10.64±0.19d | 10.26±0.74c | 10.21±0.63c |
|  | OC1 | 10.77±0.20cd | 9.65±0.81c | 11.33±0.62cd | 11.25±0.54c | 10.80±0.82c |
|  | OC2 | 11.36±0.23c | 11.06±1.12c | 12.22±0.40c | 10.36±0.38c | 11.66±0.27c |
|  | OC3 | 18.06±1.07b | 18.04±0.74b | 19.83±0.19b | 18.32±0.59b | 17.04±1.31b |
|  | OC4 | 28.23±1.93a | 29.89±0.81a | 27.76±0.37a | 26.55±0.66a | 27.78±0.46a |
| Available nitrogen (mg/kg) | OCK | 21.63±0.62c | 24.28±1.82d | 23.31±1.53a | 20.65±1.75bc | 19.05±0.09e |
|  | OC1 | 27.45±0.27b | 27.64±1.07c | 22.97±0.98a | 29.31±1.64a | 22.14±0.52d |
|  | OC2 | 28.96±1.36b | 30.17±0.44b | 21.62±1.42a | 28.69±1.10a | 28.08±0.41c |
|  | OC3 | 31.14±1.42a | 32.26±1.76a | 21.83±2.47a | 22.88±1.14b | 31.02±0.36b |
|  | OC4 | 30.98±0.39a | 31.22±0.24ab | 23.28±0.54a | 20.54±0.23c | 33.02±1.72a |
| Available phosphorus (mg/kg) | OCK | 4.67±0.23e | 5.27±0.38e | 4.03±0.72d | 4.67±0.32d | 3.23±0.15d |
|  | OC1 | 7.13±0.21d | 6.93±0.15d | 7.60±0.20c | 5.70±0.17cd | 5.40±0.44c |
|  | OC2 | 10.00±0.36c | 8.83±0.12c | 8.07±0.87c | 6.13±0.91c | 5.43±0.23c |
|  | OC3 | 13.80±0.62b | 12.87±0.40b | 11.43±0.46b | 9.50±0.61b | 7.27±0.78b |
|  | OC4 | 19.50±0.1a | 17.60±0.52a | 15.03±0.47a | 15.10±0.56a | 15.27±0.93a |
| Available potassium (mg/kg) | OCK | 83.45±4.71e | 82.26±0.74e | 79.64±4.06e | 71.67±2.73e | 68.45±2.33e |
|  | OC1 | 121.55±2.58d | 119.64±4.33d | 112.38±1.25d | 105.12±0.74d | 97.03±0.41d |
|  | OC2 | 163.57±2.23c | 158.21±5.71c | 156.31±7.05c | 133.93±5.00c | 138.69±1.61c |
|  | OC3 | 245.48±6.84b | 232.98±2.98b | 194.88±2.68b | 185.6±3.58b | 176.31±1.44b |
|  | OC4 | 316.90±3.65a | 317.86±6.19a | 291.67±14.19a | 269.64±8.18a | 266.55±20.97a |

Same small letter indicates no significance within same experiment at *P*=0.05.

TABLE S3 Effect of one-time biochar application seven years ago on soil properties

| **Index** | **Treatment** | **days after sowing** | | | | |
| --- | --- | --- | --- | --- | --- | --- |
|  |  | **19** | **50** | **89** | **108** | **137** |
| pH | SCK | 8.14±0.04c | 8.12±0.01b | 8.19±0.01c | 8.18±0.02c | 8.16±0.04b |
|  | SC1 | 8.20±0.06b | 8.19±0.03ab | 8.27±0.03a | 8.21±0.06bc | 8.20±0.04b |
|  | SC2 | 8.24±0.03ab | 8.21±0.01a | 8.29±0.01a | 8.28±0.02a | 8.29±0.06a |
|  | SC3 | 8.22±0.02ab | 8.24±0.02a | 8.23±0.01b | 8.26±0.04ab | 8.27±0.03a |
|  | SC4 | 8.27±0.03a | 8.18±0.09ab | 8.25±0.03bb | 8.27±0.02ab | 8.28±0.03a |
| Organic Carbon (g/kg) | SCK | 8.79±0.37c | 8.65±0.66c | 8.75±0.05d | 8.59±0.58c | 8.27±0.33c |
|  | SC1 | 9.36±0.35bc | 9.80±0.21bc | 9.36±0.06cd | 9.57±0.32bc | 9.96±0.03b |
|  | SC2 | 9.85±0.12b | 10.11±0.52b | 9.94±0.69c | 10.30±0.46b | 10.27±0.41b |
|  | SC3 | 10.16±0.23b | 10.02±0.34bc | 10.85±0.05b | 10.33±0.42b | 10.80±0.24b |
|  | SC4 | 12.21±1.00a | 12.20±0.99a | 12.17±0.64a | 11.95±1.33a | 12.15±1.13a |
| Available nitrogen (mg/kg) | SCK | 22.35±0.32d | 22.05±0.44c | 21.58±2.12c | 15.95±0.37d | 19.47±0.43bc |
|  | SC1 | 29.31±1.64bc | 28.09±0.32b | 27.16±0.56ab | 21.35±0.43c | 17.77±1.86c |
|  | SC2 | 31.74±1.13a | 30.30±0.44a | 28.89±0.57ab | 29.05±1.22b | 19.31±1.23bc |
|  | SC3 | 30.73±1.28ab | 29.40±1.62ab | 29.90±2.31a | 30.08±0.67ab | 21.44±1.42b |
|  | SC4 | 28.52±0.96c | 22.95±0.77c | 26.08±2.2b | 30.48±0.49a | 27.59±0.33a |
| Available phosphorus (mg/kg) | SCK | 3.97±0.32e | 3.60±0.70bc | 3.50±0.36b | 3.23±0.15c | 3.23±0.23d |
|  | SC1 | 4.40±0.26d | 3.90±0.20ab | 3.57±0.12b | 3.60±0.17bc | 5.23±0.29a |
|  | SC2 | 4.83±0.06c | 3.60±0.10bc | 4.77±0.23a | 5.07±0.15a | 3.70±0.2.00c |
|  | SC3 | 5.23±0.23b | 4.60±0.61a | 4.83±0.31a | 4.63±0.50a | 4.50±0.10b |
|  | SC4 | 5.80±0.10a | 3.50±0.40bc | 4.70±0.17a | 3.87±0.38b | 3.53±0.35cd |
| Available potassium (mg/kg) | SCK | 84.67±3.06e | 86.33±5.13b | 82.00±1.00d | 83.00±2.00c | 81.33±0.58d |
|  | SC1 | 94.67±2.89d | 93.67±1.53b | 93.33±2.31c | 92.33±0.58b | 92.33±0.58c |
|  | SC2 | 111.33±1.53c | 117.00±3.61a | 105.67±4.73b | 99.33±4.73ab | 98.67±2.52b |
|  | SC3 | 125.67±2.52a | 126.00±4.58a | 121.00±4.36a | 103.33±7.37a | 98.00±3.61b |
|  | SC4 | 120.33±2.52b | 123.67±10.21a | 105.67±5.69b | 105.00±6.56a | 105.67±5.69a |

Same small letter indicates no significance within same experiment at *P*=0.05.


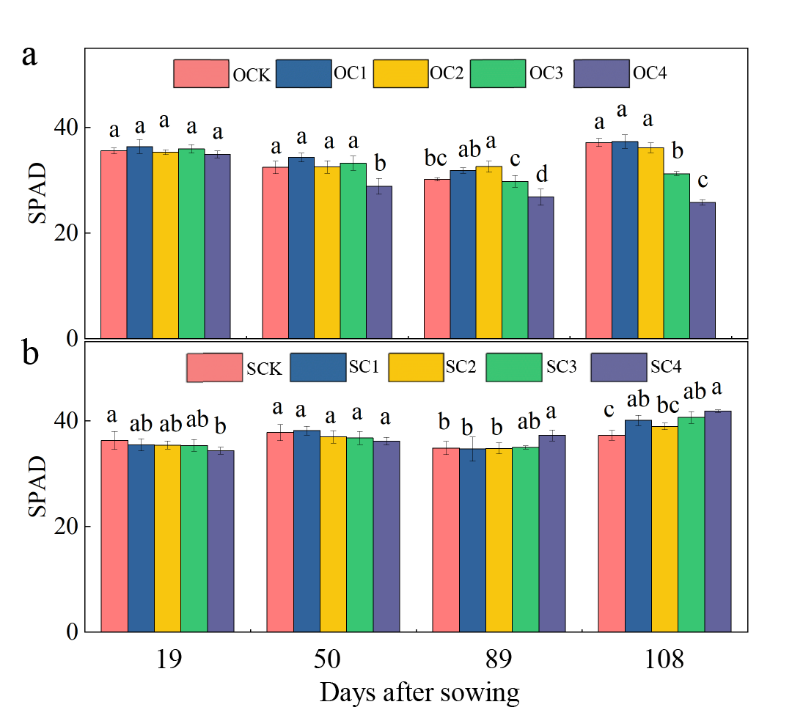


FIGURE S1 Effect of biochar application on the SPAD value of maize leaves. Same small letter indicates no significance within same experiment at *P*=0.05. (a) New biochar application treatment. (b) One-time biochar application seven years ago of treatment.


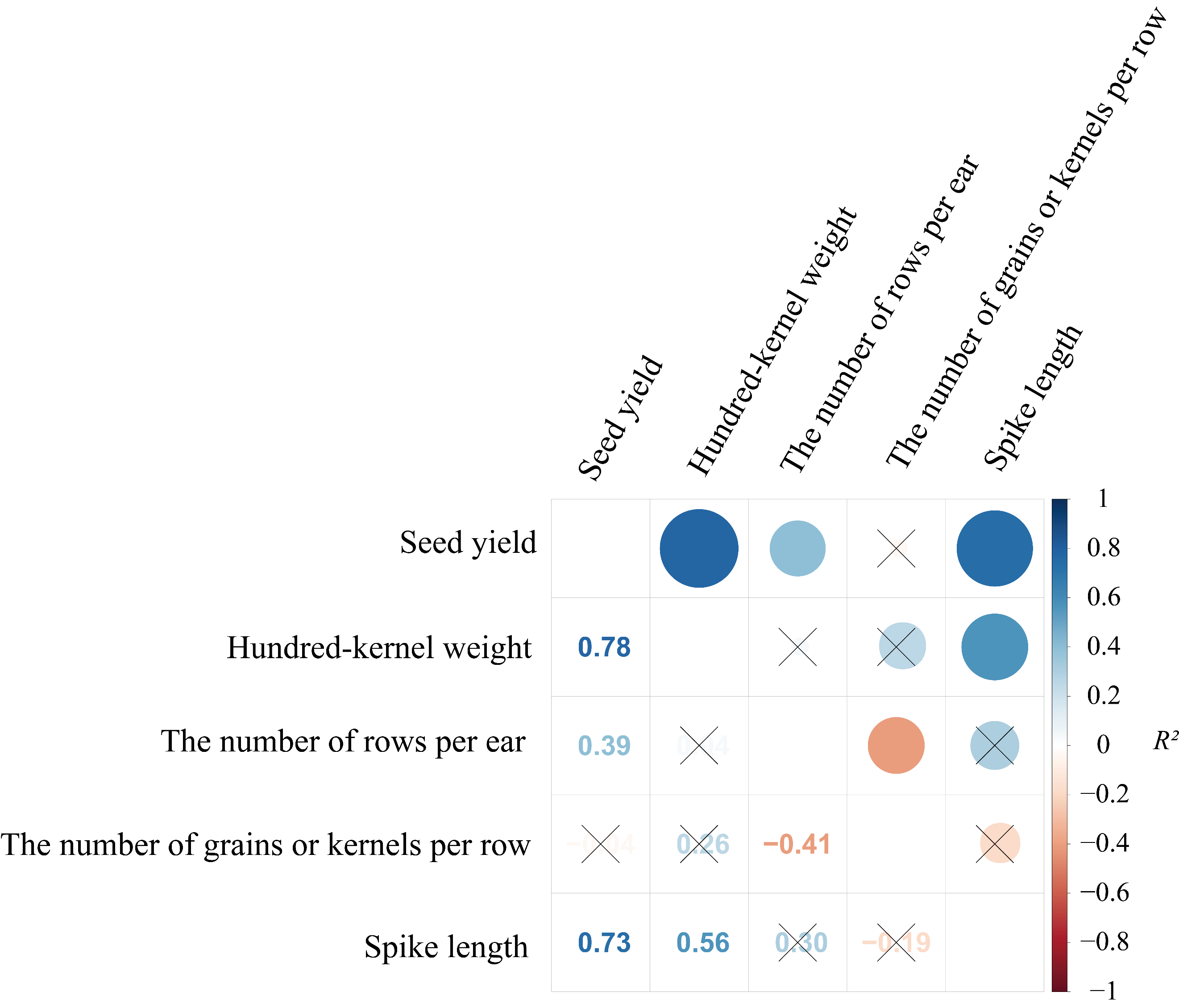


FIGURE S2 The relationship between maize yield and its components. The numbers in the figure indicate *R^2^*, and × in the figure indicates no significant correlation.
